# A Complication of Pneumothorax and Pneumomediastinum in a Non-Intubated Patient With COVID-19: A Case Report

**DOI:** 10.7759/cureus.10044

**Published:** 2020-08-26

**Authors:** FNU Sonia, Mukesh Kumar

**Affiliations:** 1 Internal Medicine, Albert Einstein College of Medicine, Bronx, USA; 2 Internal Medicine, Montefiore Medical Center, Bronx, USA

**Keywords:** covid 19, pneumothorax, pneumomediastinum

## Abstract

COVID-19 disease can lead to multiple complications such as severe acute respiratory distress syndrome (ARDS), coagulopathy, renal failure, cardiac and neurological complications. We describe a case of a patient who developed pneumothorax and pneumomediastinum in the setting of COVID-19 without having ARDS or requiring mechanical ventilation. Our patient developed sudden onset of shortness of breath and desaturation. Chest X-ray and CT chest revealed pneumothorax and pneumomediastinum. Though pneumothorax in pulmonary infection is most likely associated with increased airway pressure in acute respiratory distress syndrome and positive pressure mechanical ventilation. Pneumothorax is a life-threatening complication and if diagnosed early it can reduce mortality. In patients with COVID-19 infection, sudden clinical worsening with shortness of breath and desaturation should prompt the clinician to look for potentially treatable causes such as pneumothorax.

## Introduction

Coronavirus SARS-CoV-2 has infected more than 19 million and has caused more than 600,000 deaths to date (WHO). Initially, it was thought to be a respiratory disease only but turned out that it involves other organs as well. In one study it shows more than 15% of patients with COVID-19 are severely sick. Known complications from COVID-19 are severe acute respiratory distress syndrome, coagulopathy, renal failure, cardiac and neurological complications [1]. There are rare cases of pneumothorax and pneumomediastinum that have been reported with COVID-19 infection [2]. We describe a case report of a patient who developed pneumothorax and pneumomediastinum in the setting of COVID-19 without having acute respiratory distress syndrome (ARDS) or requiring mechanical ventilation.

## Case presentation

**Figure 1:**
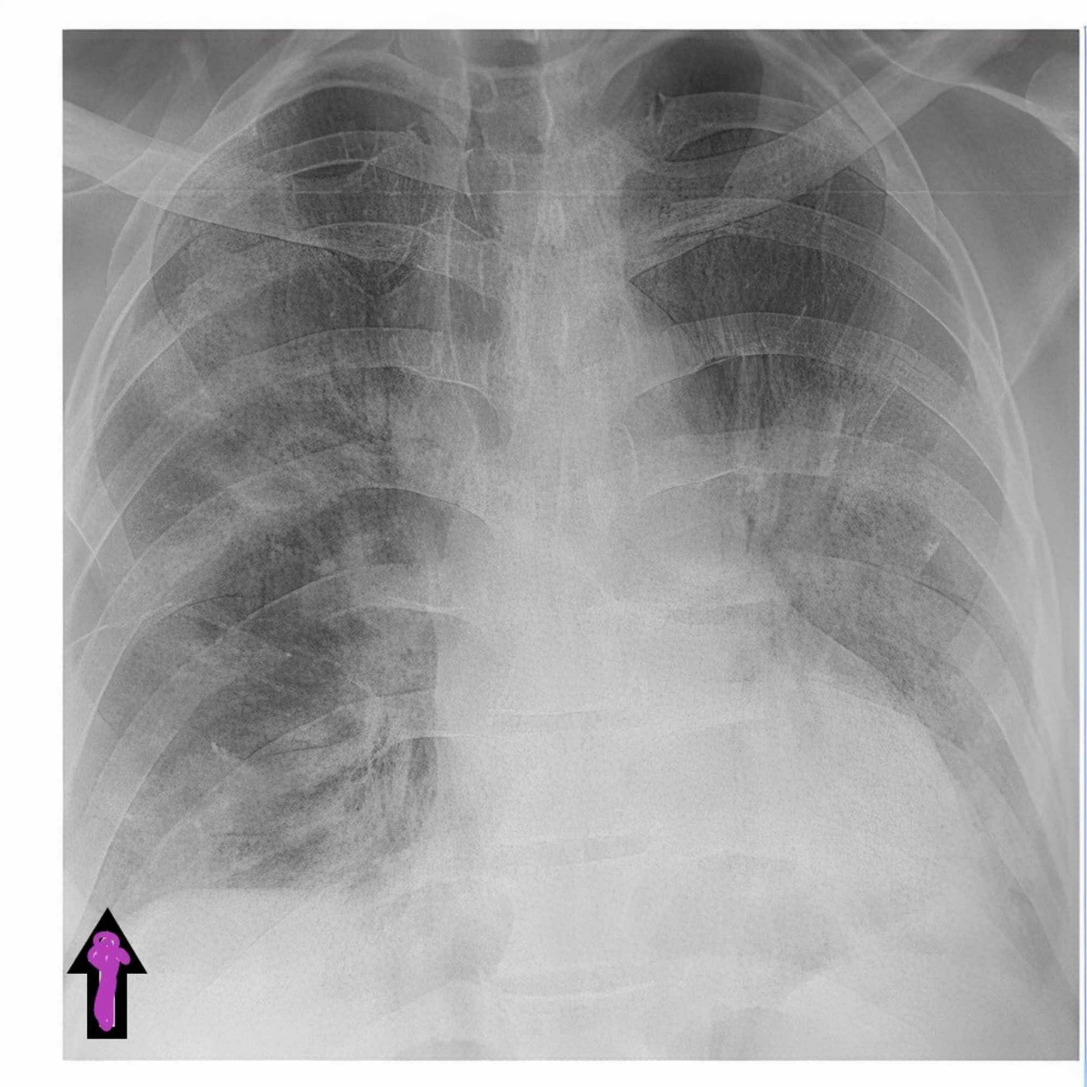
Chest X-ray on admission. Imaging reveals mild bilateral lung infiltrates. Purple arrow represents clear lung angle with no pneumothorax or pneumomediastinum.

A 62-year-old male with past medical history of diabetes mellitus, hyperlipidemia came to the emergency department with shortness of breath, fever 102 F, and dry cough started a few days before coming to the emergency department. On the initial assessment, the patient was tachycardic and dyspneic. The patient was placed on the nasal cannula 10 liters. Laboratory results revealed: COVID-19 positive, D-dimer more than 20 ug/ml, fibrinogen 852 mg/dL, ferritin 2238 ng/ml, C-reactive protein (CRP) 45.1 mg/dL, lactate dehydrogenase (LDH) 644 U/L, lymphocyte count of 0.6 k/uL. Chest X-ray on arrival showed bilateral hazy opacities. The patient was treated with methylprednisolone, hydroxychloroquine and apixaban par institutional policy. Initially, the patient required 8 L of oxygen through the nasal cannula but later on, his improved oxygen requirement came down to a 2-liter nasal cannula. On day 8, the patient complained of shortness of breath and chest tightness and dropped oxygen saturation to 84% on 2 liters. A chest X-ray was done that showed pneumothorax, pneumomediastinum, and subcutaneous emphysema. CT chest showed pneumothorax, extensive pneumomediastinum and subcutaneous emphysema. The patient was placed on non-rebreather 100% 15 L and transferred to ICU for monitored care. The patient stayed in the ICU for three days. The oxygen requirement came down and a subsequent chest X-ray revealed improving pneumothorax and pneumomediastinum (Figure 1). The patient did not require a chest tube.

**Figure 2:**
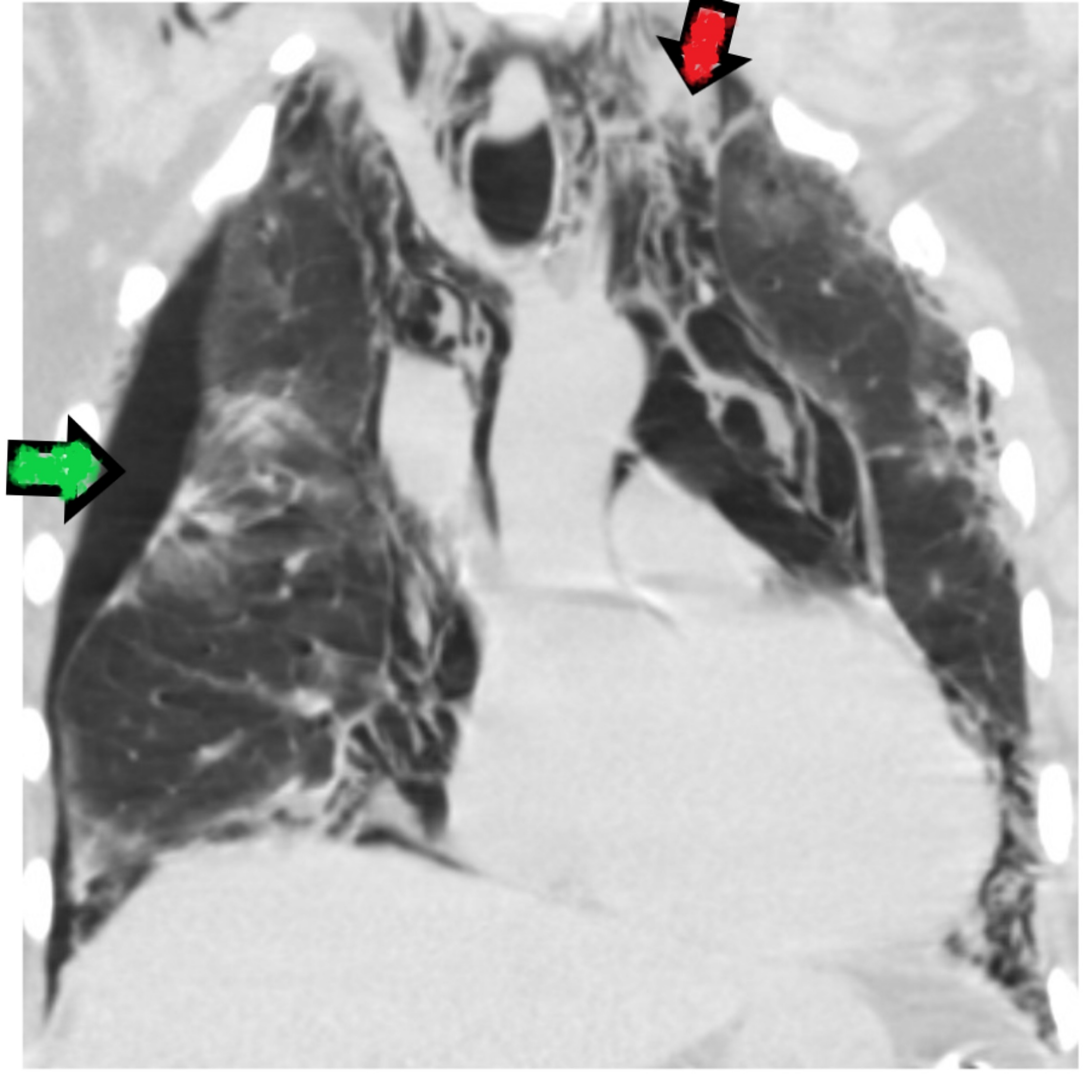
Computed tomographic image of chest on day 8 of admission. Green arrow represents pneumothorax and red arrow represents pneumomediastinum.

Chest X-ray obtained at admission revealed infiltrates in both lungs, no pleural effusion, pneumothorax or pneumomediastinum. On day 8 of admission, CT scan of chest revealed pneumothorax marked by green arrow and pneumomediastinum marked by red arrow (Figure 2).

## Discussion

Pneumothoraces can be spontaneous, iatrogenic, and traumatic. Spontaneous pneumothorax most commonly occurs in tall, thin males aged 10-30 years; some cases are associated with smoking. Iatrogenic pneumothorax is related to invasive diagnostic and treatment methods, such as transthoracic needle aspiration, subclavian vein catheterization, thoracentesis, transbronchial lung biopsy, pleural biopsy, and mechanical ventilation [3]. In some cases, pneumothoraces have been associated with ARDS in mechanically ventilated patients either from barotrauma or increased airway pressure due to ARDS [4,5]. Pneumothoraces that developed in patients with COVID-19 and ARDS have been attributed to the same etiologies. Our patient initially presented with bilateral infiltrates and required oxygen support with 8 L by nasal cannula. After treatment, he felt better and his oxygen requirement came down to 2 L. However, on hospital day 8, he suddenly deteriorated clinically with chest tightness, shortness of breath, and desaturation. Our patient did not have ARDS based on criteria and was not intubated. He had severe COVID-19 disease based on elevated markers (e.g., D-dimer, ferritin, CRP). The patient was treated with steroid therapy based on institutional policy for severe disease based on these makers.

As mentioned above, the most common causes of pneumothoraces in respiratory infection are due to very high plateau pressure from ARDS or barotrauma from mechanical ventilation. It is unclear why our patient developed pneumothorax without these conditions. It has been observed that coronavirus SARS-CoV-2 infection leads to coagulopathy with increased D-dimer and thrombotic/embolic complications such as pulmonary embolism. In one study, patients with COVID-19 had pulmonary embolism diagnosed by a CT scan of the chest [6]. Lung biopsies of patients who died from COVID revealed diffuse alveolar damage [7]. Both pulmonary embolisms and diffuse alveolar damage can lead to tissue necrosis and air leak, which can cause pneumothorax. Also, pneumothorax has been seen in patients with necrotizing bacterial pneumonia due to tissue necrosis and air leak. Our patient had coagulopathy with elevated D-dimer and diffuse bilateral infiltrates, which could have led to tissue damage, necrosis, and ultimately air leak. Our patient’s pneumothorax improved as his COVID-19 infection improved. Though he did not develop a tension pneumothorax, he did require active intervention for rapidly worsening saturation. Pneumothoraces can be fatal and early recognition is essential in order to prevent a major catastrophe. Patients' clinical status tends to deteriorate rapidly in COVID-19 infection and in some patients, there is a bimodal presentation where the patient improves clinically and then deteriorates a few days later, as in our patient.

## Conclusions

Most patients with COVID-19 present with bilateral infiltrate and ground-glass opacities. It is important to consider the diagnosis of pneumothorax in COVID-19 patients, if there is a sudden increase in work of breathing, decreased oxygen saturation, or the patient complains of chest tightness. In case of sudden deterioration, urgent chest X-ray (CXR) or bedside point-of-care ultrasound should be done to evaluate for lung sliding, and expert help should be sought.
